# The PREVASC study: *P*rospective *RE*gistry of *V*alve disease in *A*symptomatic Italian elderly *S*ubje*C*ts

**DOI:** 10.1007/s40520-025-02937-5

**Published:** 2025-03-20

**Authors:** Nazario Carrabba, Mattia Alexis Amico, Gherardo Busi, Matteo Vannini, Filippo Bruscoli, Salvatore Fortunato, Luciano Arcari, Emilio Di Lorenzo, Giampaolo Luzi, Francesco Clemenza, Francesco Amico, Giuseppe Pes, Marco Merlo, Gianfranco Sinagra, Giovambattista Desideri, Francesco Vetta, Alessandro Mugelli, Niccolo Marchionni, Alessandro Boccanelli, the PREVASC Working Group, Italian Society of Geriatric Cardiology (SICGe), Paolo Vitillo, Federico Nicastro, Davide Bonadies, Giuseppe Caliendo, Giovanna Carpentieri, Immacolata Esposito, Loreto Cedrone, Luca Arcari, Barbara Pala, Priscilla Tifi, Edoardo Cittadini, Enrico Rathina Raj, Dario Giaccio, Gabriele Di Gesaro, Salvo Storniolo, Sebastiano Puglisi, Giuseppe Leggio, Fabio Dipasqua, Caterina Di Guardo, Paola Tribulato, Emanuela Biondi, Margherita Drago, Laura Salemi, Marinella Paratore, Nicola Campana, Alessandra Gioi, Simone Angius, Accalai Emanuele, Lionti Fabio, Roberta Piras, Lina Manzi, Alberto Guarnaccia, Marco Cittar, Maddalena Rossi, Lisa Pellin, Enzo Merro, Carla Indennidate, Stefano Contessi, Anna Reginato, Ambra Fabbro, Teresa Capovilla, Francesco Venturelli, Irena Tavčar, Berardini Elvasio, Maceroni Cinzia, Murzilli Romina, Occhiuzzi Enrico, Ricci Pierluigi, Tiburzi Flavio, Carla Andrea, Lorenzini Beatrice, Palazzolo Martina

**Affiliations:** 1 Cardio-Thoracic-Vascular Department, A.O.U Careggi, Florence, Italy; 2 A.R.C.A. (Regional Associations of Outpatient Cardiologists), Rome, Italy; 3 Medical-Surgical Department of the Heart and Blood Vessels, San Giuseppe Moscati Hospital, Avellino, Italy; 4 Cardiovascular Department, San Carlo Hospital, Potenza, Italy; 5 Cardiology Unit of ISMETT (Mediterranean Institute for Transplantation and Advanced Specialized Therapies), Palermo, Italy; 6 Cardiology Unit, AOE Cannizzaro, Catania, Italy; 7 Binaghi Hospital, Cagliari, Italy; 8 Cardio-Thoracic-Vascular Department, A.S.U.G.I, Trieste, Italy; 9 Univeristy of Trieste, Trieste, Italy; 10 Geriatrics and Long-Term Care Unit, Avezzano District Hospital, Avezzano, Italy; 11 Department of Clinical, Internal, Anesthesiologic and Cardiovascular Sciences, Sapienza University of Rome, 00161 Rome, Italy; 12 Department of Experimental and Clinical Medicine, University of Florence, Florence, Italy; 13 Unicamillus University, Rome, Italy

**Keywords:** Valvular heart disease, Elderly, Risk factors prevention, Echocardiography, Screening program, Small communities

## Abstract

**Aims:**

Valvular heart disease (VHD) is the third leading cause of cardiovascular morbidity, with its incidence and public health impact projected to increase significantly. This study adopts a novel perspective, focusing on elderly individuals residing in rural areas, highlighting the unique dynamics of small-town settings.

**Methods:**

This multicenter, observational study was conducted from May 2022 to September 2023, under the coordination of the AOU Careggi Echo Core-Lab, which managed the entire screening program. In 10 small Italian villages, each municipality facilitated the enrollment of asymptomatic individuals aged ≥ 65 years, with no prior VHD history, through voluntary participation. Participants were grouped into three age categories (65–69, 70–74, and ≥ 75 years) and underwent a thorough evaluation, including a Quality of Life (QoL) questionnaire and comprehensive echocardiographic assessment focusing on VHD detection and grading.

**Results:**

Among 1,113 participants, the prevalence and severity of VHD showed a significant increase with age (*p* < 0.0001). Remarkably, 94% of individuals aged ≥ 75 years had at least one valvular defect, with 22.5% presenting moderate or severe valvulopathy, including a prevalence of 4.8% for moderate or severe aortic valve stenosis and 7.5% for mitral regurgitation. Right-sided valvulopathies followed a similar trend, affecting 71.9% of elderly participants. QoL evaluations revealed a generally positive perceived health status, with a mean score of 77 ± 16.

**Conclusions:**

Our registry highlights that the prevalence of VHD in asymptomatic individuals over 65 years living in small Italian communities is substantial, increases with age, and is predominantly degenerative in etiology. Notably, most individuals with undiagnosed VHD perceived themselves as healthy.

## Introduction

 Valvular heart disease (VHD) ranks as the third leading cause of cardiovascular morbidity [1, 2], with its incidence and public health impact projected to increase significantly due to extended life expectancy in industrialized countries [3, 4]. The EuroHeart Survey [5] highlighted a marked rise in VHD prevalence with age, with most cases being diagnosed after 65 years [3], predominantly linked to valve degeneration associated with aging and atherosclerosis [6, 7]. Moreover, the reliance on echocardiography for precise VHD assessment may lead to an underestimation of its true prevalence and its impact on morbidity and mortality [8].

In this context, the PREVASC study (Prospective REgistry of Valve disease in Asymptomatic Italian elderly SubjeCts; ClinicalTrials.gov, ID NCT05892588) was designed to evaluate the prevalence of VHD among elderly, asymptomatic individuals living in small Italian towns within a less medicalized environment. This approach contrasts with traditional studies conducted in more urbanized areas, where large-scale screening, preventive measures, and active lifestyles often facilitate earlier detection of VHD, even in asymptomatic cases. By focusing on small communities, we aimed to capture unaltered data on the prevalence of VHD in individuals who perceive themselves as healthy.

## Methods

### Design, study population, and procedures

**Fig. 1:**
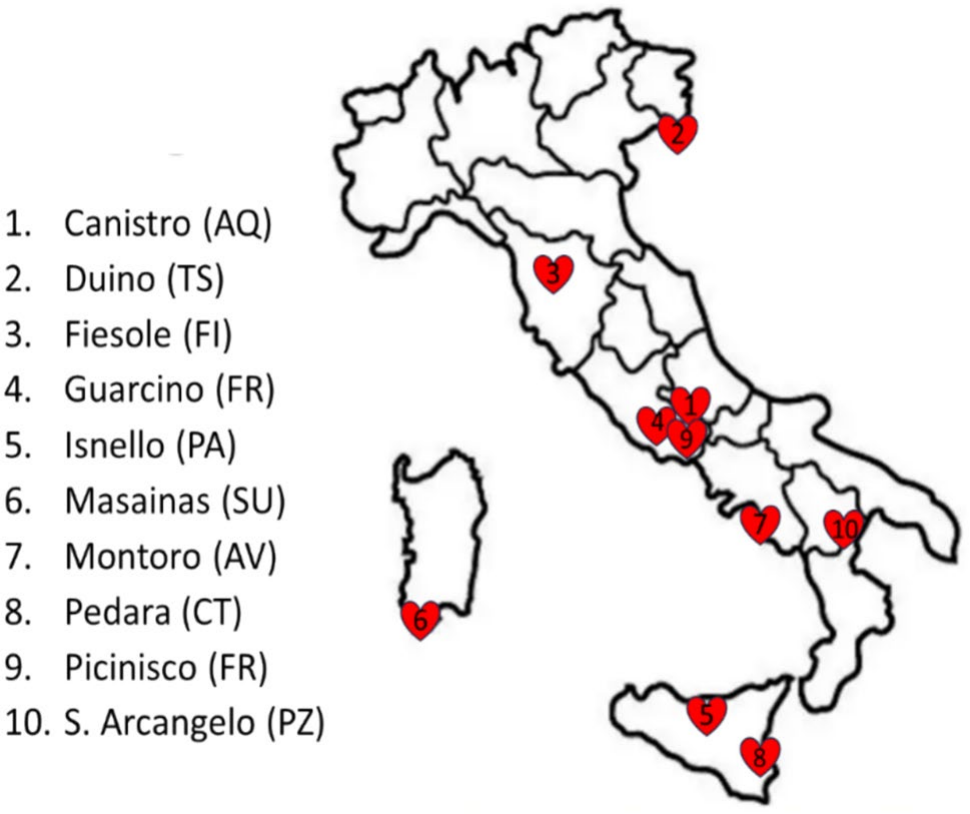
Distribution of enrolling centers across Italy

PREVASC is a multicenter, observational, cross-sectional study conducted in Italy between May 2022 and September 2023. The study was led by the AOU Careggi Hospital Echo Core-Lab, which managed study design, investigators training, data collection monitoring, and result analysis. Ten small towns (median population: 7,490 inhabitants) across northern, central, and southern Italy (Fig. 1) served as peripheral centers and enrolling sites. Although these towns were near third-level cardiological institutions, such hospitals did not act as referral centers. The protocol, endorsed by participating municipalities, utilized public health billboards to recruit subjects through voluntary participation. General practitioners (GPs) were not directly involved in recruitment. Inclusion criteria required participants to be aged ≥ 65 years, without prior VHD history, and able to provide informed consent. Prior to enrollment in the study, all subjects also had to confirm that they were asymptomatic, meaning have not symptoms that impact their everyday activities. Participants were grouped by age (65–69 years, 70–75 years, ≥ 75 years) and underwent evaluations by expert cardiologists, who provided information on VHD symptoms, misconceptions about aging, and available treatments, when indicated. Clinical and instrumental data, along with Quality of Life (QoL) information, were collected via a standardized protocol and questionnaire [9]. Anthropometric measurements, blood pressure, and heart rate were assessed following MONICA recommendations [10]. Each participant and their GP received a detailed clinical report, and those with significant VHD were referred to local cardiology centers for multidisciplinary evaluations.

The ethics Committee of Careggi University Hospital approved the study protocol, which conforms to the Declaration of Helsinki [11]. Additionally, each center obtained approval from local ethics Committees for data collection and publication. All subjects provided written, informed consent for their participation.

### Echocardiographic examinations

Echocardiographic examinations were performed by trained local physicians using commercially available machines (Vivid E 95, GE HealthCare, Little Chalfont, US) and adhering to European Society of Cardiology (ESC) guidelines [1]. All personnel received standardized training and were required to validate acquisition procedures with the Core Lab before starting. VHD was categorized as trivial/mild, moderate, or severe. Aortic valve stenosis (AS) severity was classified based on mean pressure gradients (≤ 20 mmHg for mild, > 20–39 mmHg for moderate, and ≥ 40 mmHg for severe), along with peak transaortic velocity (Vmax) and valve area (planimetric or continuity equation). Aortic regurgitation (AR) was assessed using pressure half time (PHT) or vena contracta (VC). Mitral stenosis (MS) functional area was measured via planimetric area or PHT, while mitral regurgitation (MR) was evaluated using qualitative methods (color flow mapping of the MR jet-to-left atrial area ratio) and quantitative methods, such as the Proximal Isovelocity Surface Area (PISA) method. Tricuspid regurgitation (TR) was assessed qualitatively, with PISA reserved for cases moderate/severe regurgitation. Pulmonary valvulopathy was qualitatively evaluated. Systolic pulmonary arterial pressure (PAPs) was calculated by summing the trans-tricuspid regurgitation gradient and estimated central venous pressure. Measurements were averaged from three sinus rhythm cycles or three to five atrial fibrillation (AF) cycles. Digital echocardiograms were recorded in standard DICOM format and analyzed offline by two independent expert observers at the Echo Core-Lab in Florence, who were blind to clinical data.

### Assessment of quality of life

A Quality of Life (QoL) questionnaire, based on the Italian Reference QoL data [9], was administered to participants. The questionnaire explored psychoemotional issues, including anxiety, depression, pain, and discomfort, as well as functional capacities like mobility, self-care, and performing daily activities. Each item was rated on a 1-to-5 scale, with 1 indicating severe distress or total limitation and 5 signifying optimal well-being and complete independence. Additionally, participants assessed their overall health on a visual analog scale (VAS) from 0 to 100, with 100 reflecting the best possible health state.

### Statistical analysis

Continuous variables are expressed as mean ± standard deviation (SD), while categorical variables are reported as counts and percentages. The prevalence of VHD was analyzed for the entire study population and stratified into three age groups (65–69, 70–75, and ≥ 75 years). Pearson’s chi-squared test was employed to evaluate univariable associations between demographic variables, cardiovascular risk factors, clinical history, and presence of VHD. Intra-Class Correlation (ICC) for intra- and inter-observer reliability in evaluating moderate or severe VHD cases was calculated using the Pearson correlation coefficient. Statistical analyses were conducted with SPSS (v. 29.0, Chicago, IL, USA), and a two-tailed p-value < 0.05 was deemed statistically significant.

## Results

### Baseline and medical history

**Fig. 2:**
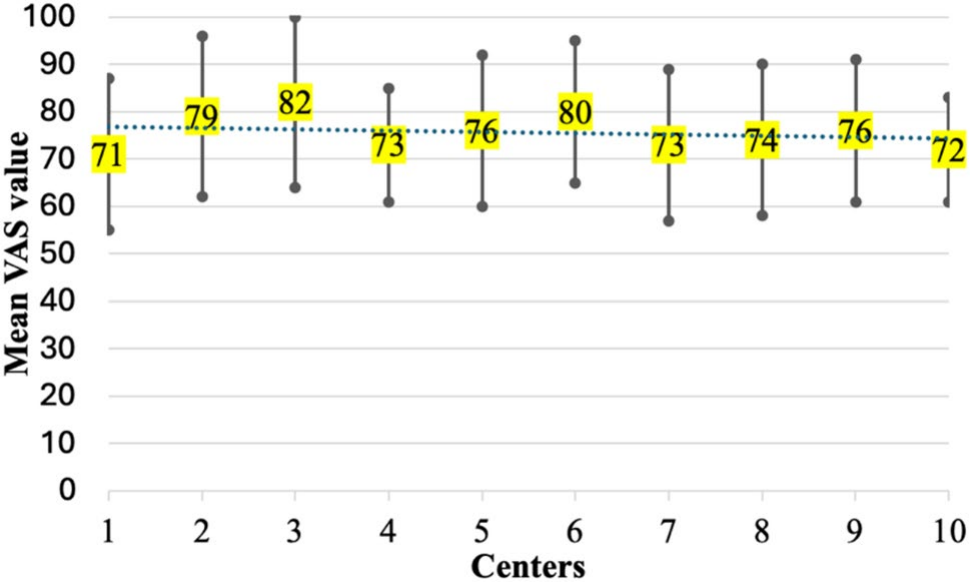
Mean Health perception VAS value in each center. VAS: visual analogue scale

The overall study population consisted of 1,113 subjects. Their baseline characteristics are showed in Table 1. Participants were evenly distributed by sex (49.9% females) and by age groups (mean: 73 ± 6 years), with 37% (*n* = 317), 33% (*n* = 367), and 30% (*n* = 334) in the 65–69, 70–75 and ≥ 75 years group, respectively. Although the large majority were in NYHA class I, 20% fell into NYHA class II or higher. The overall health perception yielded a value of 78 ± 21 on the VAS (Fig. 2), with a remarkably high mean score of 4 out of 5 in the QoL questionnaire regarding procedural capabilities. Notably, the presence of chronic pain or anxiety resulted to have a relevant negative impact, as reflected by an associated mean score of 3 out of 5 on the QoL questionnaire.

Data about demographics, cardiovascular risk factors, comorbidities, medical therapies are reported in Table 1. The most common cardiovascular risk factors were hypertension (which affected almost two thirds of participants), dyslipidemia, and diabetes. Obesity was detected in 13.8% of population. Previous percutaneous coronary intervention (PCI) and surgical coronary revascularization was detected in 4.3% and 1.9% of the population, respectively.

**Table 1.** Demographics, cardiovascular risk factors, comorbidities, and medical therapy of the study population

Demographics	
All subjects, n (%)	1113 (100%)
Male, n (%)	558 (50.1%)
Age, years	73 ± 6
65–69, years, n (%)	412 (37%)
70–74, years, n (%)	367 (33%)
≥ 75, years, n (%)	334 (30%)
Weight, kg	76 ± 15.1
Height, cm	164,4 ± 10.5
BMI	26,8 ± 4,7
BSA, m^2^	1,58 ± 0,65
Cardiovascular risk factors	
Hypertension, n (%)	708 (63.6%)
Dyslipidemia, n (%)	555 (49.9%)
Previous smoker, n (%)	332 (28.9%)
Diabetes mellitus, n (%)	221 (19.9%)
Familial history of CAD, n (%)	211 (19%)
Obesity, n (%)	154 (13.8%)
Active smoker, n (%)	126 (11.3%)
Hyperuricemia, n (%)	71 (6.4%)
Comorbidities	
COPD, n (%)	96 (8.6%)
Carotid artery disease, n (%)	93 (8.4%)
PAD, n (%)	72 (6.4%)
Chronic renal failure, n (%)	54 (4.8%)
Myocardial infarction, n (%)	48 (4.3%)
PCI, n (%)	44 (4.0%)
Anemia, n (%)	29 (2.6%)
Stroke, n (%)	22 (1.9%)
CABG, n (%)	14 (1.5%)
PCAS, n (%)	13 (1.2%)
TEA, n (%)	8 (0.8%)
PTA, n (%)	6 (0.5%)
Medical therapies	
ACE-I / ARBs, n (%)	564 (50.6%)
Statin, n (%)	392 (35.2%)
Aspirin, n (%)	256 (23.0%)
Beta-blockers, n (%)	245 (22.0%)
Other diuretics, n (%)	182 (16.4%)
PPI, n (%)	176 (15.9%)
Calcium-channel blockers, n (%)	161 (14.5%)
Antidiabetic oral agent, n (%)	148 (13.3%)
Furosemide, n (%)	52 (4.6%)
Insulin, n (%)	19 (1.7%)
MRA, n (%)	15 (1.3%)
SGLT2-i, n (%)	11 (1.0%)
Digoxin, n (%)	8 (0.7%)
ARNI, n (%)	7 (0.6%)

Continuous variables are presented as the mean ± standard deviation, while categorical variables are presented as counts and percentages

*BMI *body mass index, *BSA *body surface area, *CAD* cardiovascular disease, *COPD* chronic obstructive pneumopathy disorder, *PAD* peripheral artery disease, *CABG* coronary artery bypass graft, *PCI* percutaneous coronary intervention, *TEA* *PTA*: Percutaneous transluminal angioplasty, *CAS* carotid artery stenting, *ACE-I* Angiotensin-converting enzyme inhibitors, *ARBs* angiotensin II receptor blockers, *MRA* Mineralcorticoid receptor antagonist, *PPI* Proton pump inhibitor, *ARNI* Angiotensin receptor neprilysin inhibitor, *SGLT2-i* Sodium-glucose cotransporter 2 inhibitor

### Electrocardiographic and echocardiographic measurements

Most subjects were in sinus rhythm. However, previously unknown AF was detected in 2.7% of asymptomatic individuals.

**Fig. 3:**
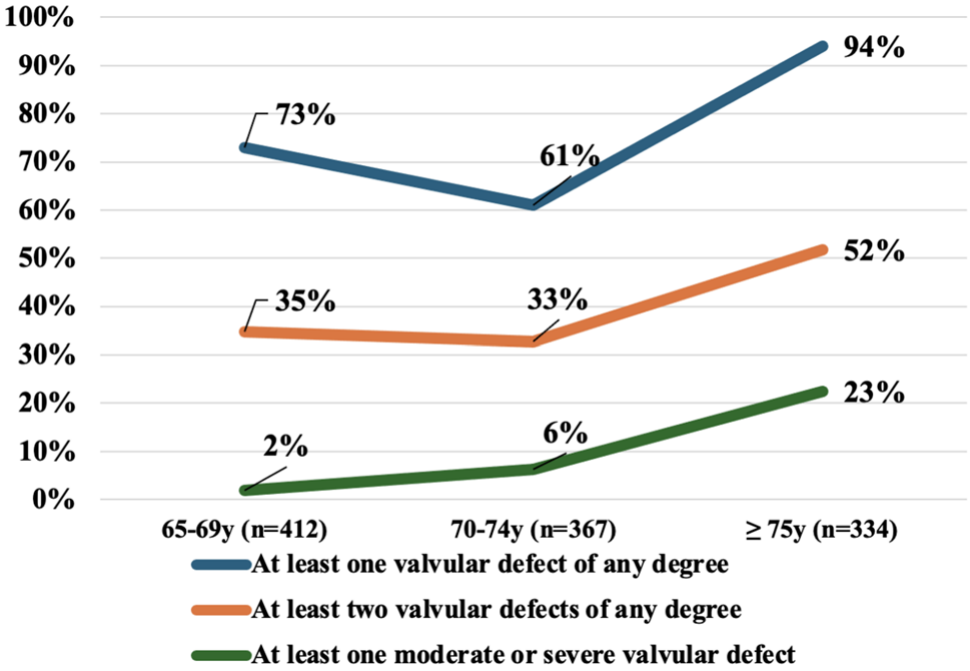
Prevalence of different valvopathies age stratified

As shown in Table 2, echocardiographic parameters were, on average, within the normal limits for either dimensions or function. Overall, some degree of VHD (either left- or right-sided) was detected in about three quarters of participants (*n* = 839, 75.4%), with a high prevalence of combined defects (*n* = 436, 39.2%; Fig. 3). A significant association was found between any VHD and some CV risk factors, such as familiarity for CAD (*p* = 0.006), diabetes (*p* = 0.02), active smoking (*p* = 0.01) and some markers of coronary artery disease such as previous myocardial infarction (*p* = 0.008) or PCI (*p* = 0.018). The prevalence of any VHD, as well as their severity (moderate or severe), increased significantly with the age (*p* < 0.0001), peaking respectively to 94.0% and to 22.5% in the ≥ 75 years group (Fig. 3). In the whole population, 52% and 61.1% showed at least one left- (L-VHD) or a right-sided VHD (R-VHD) (*p* = 0.004), being a moderate-to-severe VHD more prevalent among the L-VHD (*n* = 106 vs.15; *p* < 0.001). Four subjects had a double moderate-to-severe VHD, all in the oldest group, and all involving left-sided valves.

**Table 2.** Instrumental findings

Electrocardiogram	
ECG, n (%)	1113 (100%)
Sinus rhythm, n (%)	1067 (95.8%)
Atrial fibrillation, n (%)	30 (2.7%)
Pacemaker rhythm, n (%)	16 (1.4%)
LBBB, n (%)	30 (2.7%)
AVB, n (%)	88 (7.9%)
RBBB, n (%)	91 (8.1%)
LABB, n (%)	105 (9.4%)
LPBB, n (%)	1 (0.1%)
Echocardiografic variables	
Aortic anulus, mm	22.8 ± 4.9
Valsalva sinus, mm	32.9 ± 4.7
Sino-tubular junction, mm	28.1 ± 4.0
Ascending Aorta, mm	34.2 ± 3.9
LV end-diastolic diameter, mm	45.3 ± 5.8
LV end-systolic diameter, mm	30.2 ± 6.0
Inter-ventricular septum, mm	10.0 ± 1.7
Posterior wall, mm	9.5 ± 1.7
LV end-diastolic volume, ml	86.8 ± 25.9
LV end-systolic volume, ml	35.4 ± 14.5
Ejection fraction, %	60.1 ± 6.9
E wave, m/s	66.8 ± 22.5
A wave, m/s	84.5 ± 37.2
E/A ratio	0.8 ± 0.3
E’ lat wave, m/s	8.3 ± 3.2
E’ med wave, m/s	6.9 ± 2.9
E/E’ ratio	9.0 ± 3.1
Deceleration time, ms	230 ± 61.4
TAPSE, mm	23.0 ± 3.5
PAPs, mmHg	27.9 ± 8.9
Left atrial diameter, mm	37.1 ± 6.5
Left atrial area, cm2	18.7 ± 5.5

Continuous variables are presented as the mean ± standard deviation, while categorical variables are presented as counts and percentages

*LBBB* Left bundle branch block, *RBBB* Right bundle branch block, *AVB* atrio-ventricular block, *LABB* Left anterior bundle branch block,* LPBB* Left posterior branch block, *LV *left ventricle, *TAPSE* tricuspid anular plane systolic escursion, *PAPs* pulmonary artery pressure

### Aortic valve disease

An aortic valve disease, either stenosis or regurgitation, was present in 454 subjects (40.8%), in all cases of degenerative etiology (Table 3A). Specifically, aortic stenosis (AS) was present in 71 (6.4%) subjects, being moderate-to-severe in 29 (2.6%). Aortic regurgitation (AR) was present in *n* = 410 (36.8%) subjects, being moderate in 55 (4.9%). Severe AR degree was not found. Mixed aortic valvulopathy was detected in 27 subjects. Notably, four previously unknown severe AS were found in the oldest group. AR were associated with previous myocardial infarction (*p* = 0.006 and 0.047, respectively).

**Fig. 4:**
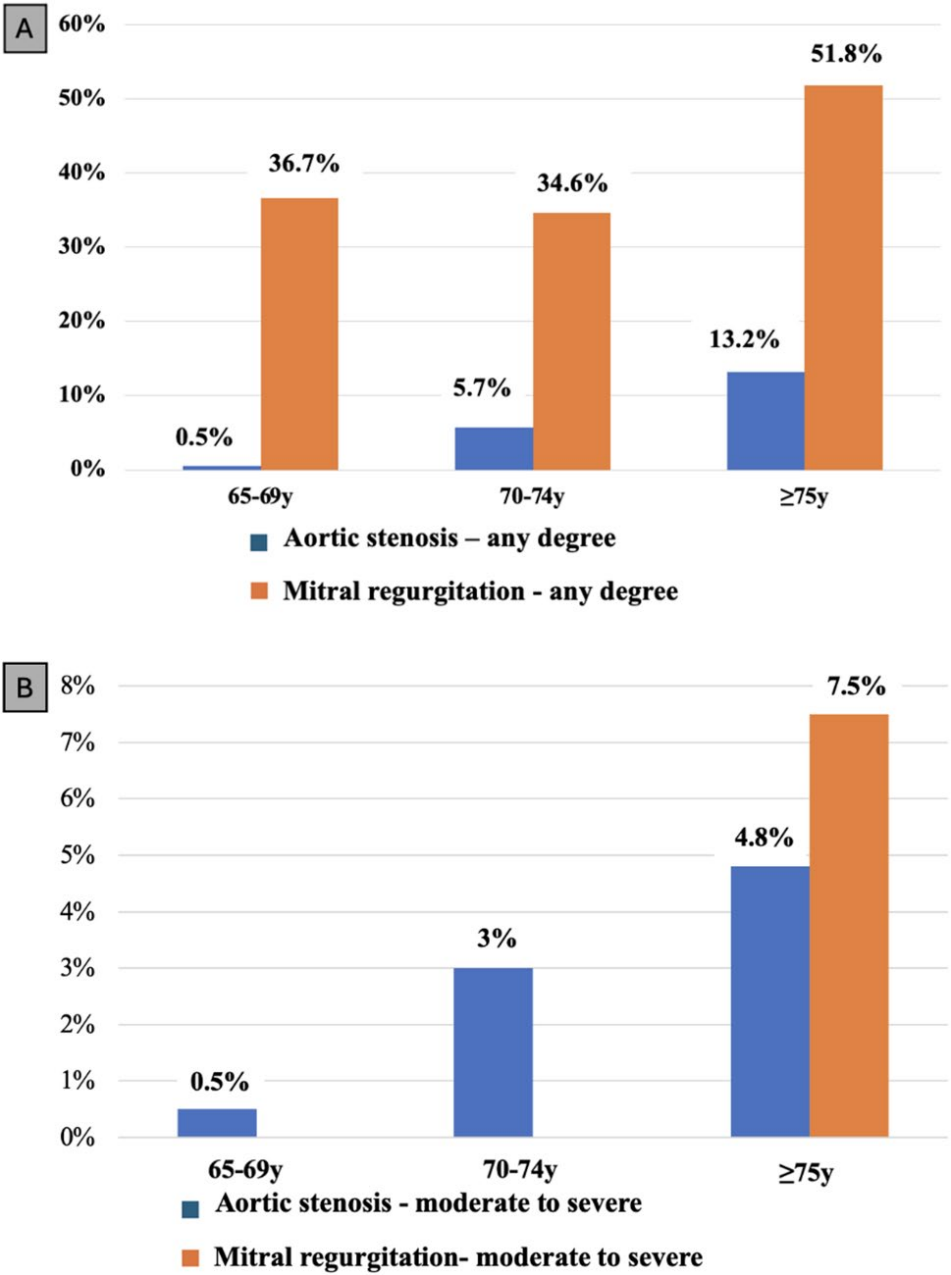
Prevalence of Aortic stenotis and Mitral Rigurgitation age stratified. Figure 4A: Prevalence of Any degree of AS and MR age stratified. Figure 4B Prevalence of Moderate or Severe AS and MR age stratified. AS: aortic stenosis. MR: mitral regurgitation

The prevalence of any type of AS and AR increased with age, and across the three age groups was 0.5%, 5.7% and 13.2% (Fig. 4A) and 27.7%, 29.1%, 56.6%, respectively.

Moreover, the prevalence of all moderate and severe AS and moderate AR increased with age and across the three age groups was 0.5%, 3.0%, 4.8% (Fig. 4B) and 1.7%, 3.3%, 10.8%, respectively.

### Mitral valve disease

In our population a mitral valve disease (Table 3A), either stenosis or regurgitation, was present in 41.6% (*n* = 464) subjects. Only 13 subjects had a mitral stenosis (MS), graded as mild and of degenerative origin in all cases. A mitral regurgitation (MR) was found in 451 (40.5%) subjects. The most common cause of MR was degenerative (87.5%), followed by functional (9.5%), and mixed (3.0%). The prevalence of any degree of mitral regurgitation increased with age, and across the three age groups was 36.7%, 34.6%, 51.8% (Table 3A *and* Fig. 4A). Notably, all moderate MR (*n* = 25) were identified in the group ≥ 75 years of age (Fig. 4B) and was closely associated with AF (*p* = 0.004). No severe MR was found.

### Right sided valvulopathies

Some degree of tricuspid regurgitation (TR; Table 3B) was found in 667 subjects (60.0%), of primary origin in two thirds (*n* = 405, 60.7%). In the remaining 262 cases (39.3%), a secondary cause was identified. The prevalence of TR increased with age. TR was graded more than mild in 15 patients (1.4%), 13 of them were found in the ≥ 75y group. No severe TR was detected. Almost the half (*n* = 310) of subjects with a TR had an associated MR, representing the 27.9% of the whole population. Pulmonary regurgitation (PR) was found in 258 subjects (23.2%), with 150 of them in ≥ 75 years group. All PR was graded as trivial/mild. No pulmonary stenosis was detected.

**Table 3:** Prevalence of valvular heart disease age stratified

	All population (*n* = 1113)	65-69y (*n* = 412)	70-74y (*n* = 367)	≥ 75y (*n* = 334)
Left sided valvular heart disease				
Subjects with L-VHD – any degree: n (%)	*578 (52.0%)*	*178 (43.2%)*	*140 (38.2%)*	*213 (63.8%)*
Aortic valve disease (A-VD)				
Subjects with A-VD – any degree; n (%)	*454 (40.8%)*	*126 (30.6%)*	*171 (46.6%)*	*211 (63.2%)*
Subjects with double A-VD – any degree; n (%)	*27 (0.4%)*	*2 (0.5%)*	*6 (1.6%)*	*19 (5.7%)*
• Aortic stenosis (AS)				
Subjects with AS – any degree; n (%)	*71 (6.4%)*	*6 (0.5%)*	*21 (5.7%)*	*44 (13.2%)*
Trivial/mild AS; n (%)	*42 (3.8%)*	*4 (0.7%)*	*10 (2.7%)*	*28 (8.4%)*
Moderate AS; n (%)	*25 (2.2%)*	*2 (0.5%)*	*11 (3.0%)*	*12 (3.6%)*
Severe AS; n (%)	*4 (0.4%)*	*0 (0%)*	*0 (0%)*	*4 (1.2%)*
• Aortic regurgitation (AR)				
Subjects with AR – any degree; n (%)	*410 (36.8%)*	*114 (27.7%)*	*107 (29.1%)*	*189 (56.6%)*
Trivial/milt AR; n (%)	*355 (31.9%)*	*108 (26.2%)*	*91 (24.8%)*	*156 (46.7)*
Moderate AR; n (%)	*55 (4.9%)*	*7 (1.7%)*	*12 (3.3%)*	*36 (10.8%)*
Severe AR; n (%)	*0 (0%)*	*0 (0%)*	*0 (0%)*	*0 (0%)*
Mitral valve disease (M-VD)				
Subjects with M-VD – any degree; n (%)	*459 (41.2%)*	*159 (38.6%)*	*121 (33.0%)*	*179 (53.6%)*
Subjects with double M-VD – any degree; n (%)	*5 (0.4%)*	*0 (0%)*	*0 (0%)*	*5 (1.5%)*
• Mitral stenosis (MS)				
Subjects with MS – any degree; n (%)	*13 (1.16%)*	*3 (0.7%)*	*4 (1.1%)*	*6 (1.8%)*
• Mitral regurgitation (MR)				
Subjects with MR – any degree; n (%)	*451 (40.5%)*	*151 (36.7%)*	*127 (34.6%)*	*173 (51.8%)*
Trivial/mild MR (n, global %)	*426 (38.3%)*	*153 (37.1%)*	*116 (31.6%)*	*156 (46.7%)*
Moderate MR (n, global %)	* 25 (2.2%)*	*0 (0%)*	*0 (0%)*	*25 (7.5%)*
Severe MR (n, global %)	*0 (0%)*	*0 (0%)*	*0 (0%)*	*0 (0%)*
Right sided valvular heart disease				
Subjects with R-VHD – any degree: n (%)	*680 (61.1%)*	*246 (59.7%)*	*193 (52.6%)*	*240 (71.9%)*
•Tricuspidal regurgitation (TR)				
Subjects with TR – any degree; n (%)	*667 (60.0%)*	*240 (58.3%)*	*207 (56.4%)*	*220 (65.9%)*
Trivial/mild TR (n, global %)	*652 (58.6%)*	*239 (58.1%)*	*206 (56.1%)*	*207 (62.0%)*
Moderate TR (n, global %)	*15 (1.4%)*	*1 (0.2%)*	*1 (0.3%)*	*13 (3.9%)*
Severe TR (n, global %)	*0 (0%)*	*0 (0%)*	*0 (0%)*	*0 (0%)*
•Pulmonary regurgitation (PR)				
Subjects with PR – any degree; n (%)	*258 (23.2%)*	*52 (12.6%)*	*56 (15.3%)*	*150 (44.9%)*
Trivial/mild PR (n, global %)	*258 (23.2%)*	*52 (12.6%)*	*56 (15.3%)*	*150 (44.9%)*
Moderate PR (n, global %)	*0 (0%)*	*0 (0%)*	*0 (0%)*	*0 (0%)*
Severe PR (n, global %)	*0 (0%)*	*0 (0%)*	*0 (0%)*	*0 (0%)*

*L-VDH *Left sided valvular heart disease, *R-VHD *Right sided valvular heart disease

### Reliability of echocardiographic assessment of VHD severity

Intra- and inter‐observer reproducibility of moderate and severe VHD was assessed in all subjects. The intra‐observer, intra‐class correlation coefficients (rho) for VHD severity was 0.96 (95% CI = 0.94–0.98) and 0.98 (95% CI 0.97–0.99). The inter‐observer, intra‐class correlation coefficient (rho) for VHD severity was 0.95 (95% CI 0.93–0.97).

## Discussion

The main findings of our study can be summarized as follows:

1. The prevalence of any VHD in subjects older than 65 years is substantial.
2. The prevalence and the severity of any VHD increase with age.
3. The most prevalent etiology of VHD is degenerative.

The relationship between aging of the population and the prevalence and severity of VHD aligns with established pathophysiological evidence and findings from other European registries [8, 12–15].

According to the Euro Heart Survey on Valvular Disease, aortic valve disease is the most prevalent VHD in developed countries, predominantly of degenerative origin [3, 16]. Notably, approximately two-thirds of all VHD manifest in subjects over 75 years of age [17, 18]. Our findings substantiate and reinforce these results, emphasizing both the prevalence and etiology of aortic valve disease. Consistent with previous observations, our study reveals a prevalence of moderate-to-severe AS of 4.8% in subjects aged ≥ 75years. This finding is particularly significant given the current European guidelines recommending Transcatheter Aortic Valve Implantation (TAVI) for high-risk patients aged 75 years and older [19]. The demographic shift towards an aging population highlights the importance of addressing the healthcare needs of this age group, particularly given the anticipated doubling of elderly subjects with indications for treatment by 2050, in both USA and Europe [18, 20]. Consequently, the implementation of a standardized screening program for aortic valve disease becomes strategically imperative for the early detection and management of this condition. Our registry did not detect any cases of bicuspid aortic valve disease, maybe due to the high prevalence of aortic calcification that impact spatial resolution. Interestingly, high-resolution CT scans have demonstrated a notably high prevalence (over 20%) of bicuspid aortic valve stenosis among older TAVI candidates [21, 22].

AR is the fourth most common VHD globally, as highlighted in previous studies [14, 15]. In our investigation, we observed a higher prevalence of any degree of AR compared to AS, and both were associated with prior myocardial infarction, suggesting that inflammation may be a common pathway of the disease processes. Generally, AR has been linked to hypertension, particularly diastolic hypertension [23]. Thus, we acknowledge the possibility that the observed link with previous myocardial infarction may be influenced by effect of chance or other unknown factors.

Mitral valve disease is the third leading cause of VHD globally [12], while MR is the second most prevalent VHD in Europe [24]. In our study, the most common etiology of M-VD is degenerative. In addition, functional MR was less prevalent, and its occurrence was associated primarily with AF. This relationship is likely due to left atrial enlargement and mitral annular dilatation in patients with longstanding AF. Recognizing the significance of early interception in cases of high surgical risk, especially for functional MR, highlights the crucial role of early percutaneous repair for optimal patient outcomes [25–27]. This further emphasizes the importance of an early and standardized screening program for mitral valve disease and its subsequent appropriate management. In the context of chronic MR, it is notable that the prognosis for severe, degenerative MR is poor. However, timely correction of the condition is associated with a life expectancy comparable to that of the normal population [28–30].

Although its global prevalence is declining, MS is increasingly observed among elderly individuals in high-income countries [3]. However, in our study population, the prevalence of MS is notably low, highlighting a predominantly degenerative etiology. The main distinctive feature of degenerative MS is Mitral Annulus Calcification (MAC) [31, 32]. The limited migration flow in small towns of Italy may contribute significantly to these findings. This observation contrasts with trends in immigrant populations, who are more likely to have had Rheumatic Heart Disease (RHD), a condition that is typically rare among inhabitants of high-income countries [33, 34]. The interaction between demographic factors, migration patterns, and the specific etiology of MS highlights the complexity of understanding its prevalence and distribution.

TR is emerging as a growing public health concern, with over 4% of individuals over 75 years exhibiting clinically relevant TR [35–37]. Despite the rising interest, comprehensive global epidemiological data are lacking, and national screening studies indicate a variable prevalence. For instance, nearly 4% of individuals over 75 years of age in some regions have clinically relevant TR, while in UK the prevalence was 2.7% and, in China, the prevalence was only 1.1% in comparable age groups [6]. In our study, TR was predominantly graded as mild, with moderate cases notably observed in older individuals, in most cases with a primary etiology. Trace or mild TR is almost universally present and should not be regarded as a disease, since it is frequently detected in normal subjects as a collateral finding of a normal echocardiographic exam [38]. Current guidelines recommend transcatheter treatment for symptomatic patients with isolated secondary TR, without severe ventricular dysfunction or pulmonary hypertension, and at high surgical risk, as deemed by the Heart Team [1]. Consequently, the window for transcatheter treatment is currently limited. However, with the promising early success reported in the TRILUMINATE [39–41] and TRI.fr [42] trials, this approach is expected to become more diffused in the next years. Notably, the finding that 46.5% of all TR cases were associated with MR reinforces the importance of a comprehensive evaluation of both left- and right-side VHD for their optimal management [19, 43].

Results from the QoL questionnaires suggest that our study population generally perceives their health status positively and maintains a high level of functional independence in daily activities. However, the substantial number of patients in NYHA class II or higher, serves as a cautionary signal, warranting further exploration of this subset of the population. While a statistical correlation with the presence of VHD is not established, the non-specificity of NYHA classification symptoms underscores the importance of delving more deeply into this subpopulation.

Additionally, the observation of 13.8% obesity and 2.7% unknown AF in our population raises concerns and emphasizes the need for the promotion and implementation of a health policy that includes lifestyle modification and appropriate anticoagulation therapy, respectively [44]. These findings represent significant “red flags”, necessitating a proactive approach to address these health indicators and mitigate potential risks linked to undertreatment.

### Clinical implication

The novelty of our study’s approach consists of focusing on small communities, usually left unexplored in conventional epidemiological studies conducted in larger towns. In making this choice, we have tried to fill the gap in the current literature, providing a more holistic understanding of VHD within a les medicalized context. Given the expected aging population and the importance of preventing irreversible cardiac damage related to VHD, a standardized national screening echocardiographic program could be proposed. This would enable early detection of VHD and facilitate the planning of the most appropriate and effective management for individuals who otherwise consider themselves healthy.

### Limitations

Several limitations inherent to the study design are to be acknowledged. First, the multicentric registry approach, despite the presence of a core lab, introduces potential variability in screening protocols, echocardiographic data acquisition, and results interpretation across diverse sites and operators. While efforts are made to ensure consistency, the involvement of multiple operators may introduce nuances that may limit the reliability of the findings. In addition, in certain individuals, such as those with obesity or chronic lung disease, suboptimal echocardiographic imaging may occur due to low image quality or technical issues related to the misalignment of the Doppler beam with the high-velocity jet. These factors may impact the quality of valve evaluation, potentially introducing a non-modifiable bias. 1. Third, due to the cross-sectional nature of our study, no information can be provided on the evolution of VHD. Finally, due to the relatively small sample size and the population enrolled, caution is needed in generalizing our result to different populations living in different countries.

## Conclusion

Our registry demonstrated that the prevalence of VHD in asymptomatic individuals aged more than 65 years, residing in small communities’ Italian areas, is not negligible, increase with population aging, and has a degenerative etiology in most case. Moreover, it is noteworthy that the majority of individuals with unknown VHD in our study perceive themselves as healthy.

## Data Availability

The data supporting this study are confidential and are available from the corresponding author upon reasonable request and under specific conditions, subject to institutional and ethical guidelines.

## Appendix

*The Prevasc Working Group*: MONTORO (AV): Paolo Vitillo, Federico Nicastro, Davide Bonadies, Giuseppe Caliendo, Giovanna Carpentieri, Immacolata Esposito; PICINISCO (FR): Loreto Cedrone, Luca Arcari, Barbara Pala, Priscilla Tifi, Edoardo Cittadini, Enrico Rathina Raj, Dario Giaccio; ISNELLO (PA): Gabriele Di Gesaro, Salvo Storniolo, Sebastiano Puglisi, Giuseppe Leggio; PEDARA (CT): Fabio Dipasqua, Caterina Di Guardo, Paola Tribulato, Emanuela Biondi, Margherita Drago, Laura Salemi, Marinella Paratore; MASAINAS (SU): Nicola Campana, Alessandra Gioi, Simone Angius, Accalai Emanuele, Lionti Fabio, Roberta Piras; DUINO (TS): Lina Manzi, Alberto Guarnaccia, Marco Cittar, Maddalena Rossi, Lisa Pellin, Enzo Merro, Carla Indennidate, Stefano Contessi, Anna Reginato, Ambra Fabbro, Teresa Capovilla, Francesco Venturelli, Irena Tavčar; CANISTRO (AQ): Berardini Elvasio, Maceroni Cinzia, Murzilli Romina, Occhiuzzi Enrico, Ricci Pierluigi, Tiburzi Flavio, Carla' Andrea, Lorenzini Beatrice, Palazzolo Martina.
