# Correction: The PREVASC study: *P*rospective *RE*gistry of *V*alve disease in *A*symptomatic Italian elderly *S*ubje*C*ts

**DOI:** 10.1007/s40520-025-03085-6

**Published:** 2025-06-21

**Authors:** Nazario Carrabba, Mattia Alexis Amico, Gherardo Busi, Matteo Vannini, Filippo Bruscoli, Salvatore Fortunato, Luciano Arcari, Emilio Di Lorenzo, Giampaolo Luzi, Francesco Clemenza, Francesco Amico, Giuseppe Pes, Marco Merlo, Gianfranco Sinagra, Giovambattista Desideri, Francesco Vetta, Alessandro Mugelli, Niccolo Marchionni, Alessandro Boccanelli, the PREVASC Working Group, Italian Society of Geriatric Cardiology (SICGe), Paolo Vitillo, Federico Nicastro, Davide Bonadies, Giuseppe Caliendo, Giovanna Carpentieri, Immacolata Esposito, Loreto Cedrone, Luca Arcari, Barbara Pala, Priscilla Tifi, Edoardo Cittadini, Enrico Rathina Raj, Dario Giaccio, Gabriele Di Gesaro, Salvo Storniolo, Sebastiano Puglisi, Giuseppe Leggio, Fabio Dipasqua, Caterina Di Guardo, Paola Tribulato, Emanuela Biondi, Margherita Drago, Laura Salemi, Marinella Paratore, Nicola Campana, Alessandra Gioi, Simone Angius, Accalai Emanuele, Lionti Fabio, Roberta Piras, Lina Manzi, Alberto Guarnaccia, Marco Cittar, Maddalena Rossi, Lisa Pellin, Enzo Merro, Carla Indennidate, Stefano Contessi, Anna Reginato, Ambra Fabbro, Teresa Capovilla, Francesco Venturelli, Irena Tavčar, Berardini Elvasio, Maceroni Cinzia, Murzilli Romina, Occhiuzzi Enrico, Ricci Pierluigi, Tiburzi Flavio, Carla Andrea, Lorenzini Beatrice, Palazzolo Martina

**Affiliations:** 1 Cardio-Thoracic-Vascular Department, A.O.U Careggi, Florence, Italy; 2 A.R.C.A. (Regional Associations of Outpatient Cardiologists), Rome, Italy; 3 Medical-Surgical Department of the Heart and Blood Vessels, San Giuseppe Moscati Hospital, Avellino, Italy; 4 Cardiovascular Department, San Carlo Hospital, Potenza, Italy; 5 Cardiology Unit of ISMETT (Mediterranean Institute for Transplantation and Advanced Specialized Therapies), Palermo, Italy; 6 Cardiology Unit, AOE Cannizzaro, Catania, Italy; 7 Binaghi Hospital, Cagliari, Italy; 8 Cardio-Thoracic-Vascular Department, A.S.U.G.I, Trieste, Italy; 9 Univeristy of Trieste, Trieste, Italy; 10 Geriatrics and Long-Term Care Unit, Avezzano District Hospital, Avezzano, Italy; 11 Department of Clinical, Internal, Anesthesiologic and Cardiovascular Sciences, Sapienza University of Rome, 00161 Rome, Italy; 12 Department of Experimental and Clinical Medicine, University of Florence, Florence, Italy; 13 Unicamillus University, Rome, Italy

**Correction: Aging Clinical and Experimental Research (2025) 37:98** 10.1007/s40520-025-02937-5.

In this article the consortium member

"PREVASC Working Group, Italian Society of Geriatric Cardiology (SICGe)" was misspelt as PREVASC Working Group, Italian Society of Geriatrica Cardiology (SICGe)”

The original article has been corrected.

